# Incident mental disorders following cancer: analysis of real-world psychiatric outcomes in a nationwide population-based cohort study in Denmark across three decades

**DOI:** 10.1038/s41416-026-03460-8

**Published:** 2026-04-29

**Authors:** Franziska Springer, Mette Kielsholm Thomsen, Jorne Biccler, Lars Vedel Kessing, Anja Mehnert-Theuerkauf, Annika von Heymann, Christoffer Johansen

**Affiliations:** 1 Department of Medical Psychology and Medical Sociology, Comprehensive Cancer Center Central Germany (CCCG), University Medical Center Leipzig, Leipzig, Germany; 2 Department of Clinical Epidemiology, Aarhus University and Aarhus University Hospital, Aarhus, Denmark; 3 Sanos, Søborg, Denmark; 4 Psychiatric Center Copenhagen, Frederiksberg, Copenhagen Denmark; 5 Department of Clinical Medicine, University of Copenhagen, Copenhagen, Denmark; 6 Cancer Late Effects Research, Department of Oncology, Centre for Cancer and Organ Diseases, Rigshospitalet, Copenhagen, Denmark

**Keywords:** Cancer, Epidemiology, Psychiatric disorders

## Abstract

**Background:**

Cancer and mental disorders may influence one another, yet research on the risk of developing a new-onset mental disorder following cancer, other than depression, remains limited.

**Methods:**

In a nationwide register-based study, patients were followed from incident cancer diagnosis between 1995 and 2015 until end of follow-up 2023, excluding those with preexisting mental disorders. Patients were matched with cancer-free individuals on age, sex, socioeconomic position and comorbidities. We estimated incidence rates (IR) of mental disorders through psychiatric diagnoses and psychotropic medication prescriptions, as well as hazard ratios (HR) in comparison to cancer-free individuals.

**Results:**

We included 289,391 cancer patients and 1,031,057 population-matched cancer-free comparisons. Across the cancer cohort, 116,118 developed any incident mental disorder, with a HR of 2.3 [95%-CI: 2.3–2.3] and varying rates across tumor types. IR and HR were highest in the first year after cancer diagnosis and decreased rapidly thereafter, yet the HR remained elevated exceeding ten years. Highest IRs and HRs were observed for anxiety, depression and substance use disorders. Our results were confirmed by several sensitivity analyses.

**Conclusion:**

The incidence and risk elevation of incident mental disorders in cancer patients vary based on sex, cancer type, time since diagnosis and type of mental disorder.

## Introduction

Cancer and mental disorders represent two of the most profound stigmas in life, each capable of affecting all aspects of an individual’s life and contributing to a decline in somatic and psychological function and social isolation [1–3]. As cancer rates continue to rise globally [4] and the vast majority of citizens are treated for a mental disorder during their lifetime [5], these two conditions pose growing challenges for healthcare systems worldwide.

Although the link between cancer and mental disorders is well-documented, with prevalence rates of mental disorders in oncological populations at around 30% [6–9], the downstream impact of cancer on the development of incident mental disorders remains poorly understood. The incidence, however, varies depending on the data source, such as clinical diagnostic interviews, hospital admissions for mental disorders, or prescriptions of psychotropic medication [5, 7, 10, 11]. To draw valid conclusions for clinical practice, population-based longitudinal studies are needed, reflecting real-world psychiatric outcomes in non-biased samples with minimal loss to follow-up. Previous population-based research based on data of psychiatry registries consistently shows that cancer patients face an elevated risk of developing depression compared to the general population [9, 12–15]. However, evidence on mental disorders other than depression remains limited to date [10, 16], and results on less common mental disorders, such as bipolar disorder, schizophrenia, or posttraumatic stress disorder are lacking completely. In addition, previous population-based studies have primarily focused on common cancers such as breast, prostate, colorectal or lung cancer, and no study has comprehensively stratified the incidence rates and risks of mental disorders across all common and rare tumor types.

Importantly, comorbid mental disorders such as depression or anxiety can adversely impact cancer treatment adherence, prolong hospital stays, and increase cancer-related mortality [11, 17–19]. This underscores the critical importance of prevention, early detection and targeted treatment of mental disorders in oncological populations.

The aim of this study is to contribute to a better understanding of real-world, new-onset mental disorders in cancer patients. We present incidence estimates of mental disorders, identified through psychiatric diagnoses and prescriptions of psychotropic medication, in a nationwide population-based cohort of patients with incident cancer across all tumor types, and compare estimates to those in a matched, cancer-free cohort from the general population.

## Methods

### Study design and cohort

In this population-based cohort study, we followed adult patients from the date of their primary cancer diagnosis between 1 January 1995 and 31 December 2015, excluding non-melanoma skin cancer, until end of follow-up 1 January 2023. Patients were aged 18 years or older at the time of cancer diagnosis. To identify a cancer-free comparison cohort from the general population, we matched each patient with up to five comparisons – as possible – on age, sex, index date, socioeconomic position and physical comorbidity (Charlson Comorbidity Index [20], CCI). If no comparisons were available, the patient was excluded. Individuals with a prior cancer diagnosis, or with a mental disorder before their cancer diagnosis or index date were excluded.

Patients and comparisons were followed until the first occurrence of a mental disorder of interest, death (competing risk), emigration (censoring), a primary cancer diagnosis in the comparison cohort (competing risk), or end of follow-up (censoring).

This register-based study is in accordance with Danish regulations complying with the General Data Protection Regulation of the EU. The study was reported to the Danish Data Protection Agency through institutional registration at Copenhagen University Hospital (p-2023-14473). Informed consent from cohort members and ethical approval are not required for using Danish registry data in observational studies.

### Data sources and variables

We retrieved data from national registries and linked data using the universal personal registration number [21] assigned to all residents at birth or immigration since 1 April 1968. We identified our study cohort of primary cancer patients using data from the Danish Cancer Registry [22]. The Danish National Patient Registry [23] was used to assess the modified CCI, excluding cancer. Information on sex, age, death, emigration/immigration and cohabitation status were obtained from the Danish Civil Register [21]. Data on education, employment and income at the time of cancer diagnosis or index date were obtained from Danish nationwide administrative social registries [24, 25] by Statistics Denmark.

The primary outcome was incident mental disorder defined as a combination of two measures: (1) a mental disorder diagnosis registered as an inpatient or outpatient hospital admission – capturing more severe cases –, or (2) prescription of relevant psychotropic medication, which can be prescribed by general practitioners or privately practicing psychiatrists – capturing mental disorders with higher sensitivity. This was assessed by ICD-10 codes from the Danish National Patient Psychiatry Registry, that contains data on all admissions to psychiatric inpatient and outpatient facilities, and Anatomical Therapeutic Chemical Classification (ATC) from the Danish National Prescription Registry [26] for redeemed prescriptions of psychotropic medication.

Mental disorder diagnoses included ICD-10 codes F10-F79: substance use disorders, schizophrenia and related disorders, bipolar disorders, unipolar depression, anxiety disorders, posttraumatic stress disorder, adjustment disorder, neurotic and somatoform disorders, behavioral syndromes associated with physiological disturbances and physical factors, personality disorders, and other psychiatric disorders. Psychotropic medication comprised antipsychotics, antidepressants, lithium, anxiolytics, and medication for alcohol and opioid dependence (Supplement S1). We excluded F00-F09, i.e., dementia, delirium and other organic related mental disorders, and F80-F98, i.e., developmental disorders, as well as medications for sleep, dementia, neurological disorders, smoking cessation, and ADHD, to avoid overestimating mental disorders.

We divided all patients into 28 diagnostic groups of the most common cancer types and one group with various very rare cancers (Supplement S2), to stratify results by cancer type.

### Statistical analyses

First, incidence rates (IR) per 1000 person-years with 95% confidence intervals (CI) were calculated for any mental disorder, different types of mental disorders and medication classes, stratified by sex. To provide valid data for types of mental disorders, patients were not censored following their first mental disorder of interest to ensure that subsequent diagnoses within other subgroups of mental disorders could be recorded. The cumulative incidence of any mental disorder in the cancer cohort was estimated as the risk 1-year to 25-years following the cancer diagnosis. We then applied Cox proportional hazard models to estimate hazard ratios (HR) for mental disorders with corresponding 95%-CIs, using age as the underlying time-scale. The Cox models were not adjusted further, as they included weights to account for not always having five comparisons and robust standard errors to account for the clustering induced by the matching on sociodemographic confounders. Subsequently, we stratified results by cancer type.

Several sensitivity analyses were conducted to address influencing factors: 1) To increase specificity, we applied stricter criteria for psychotropic medication, as applied in our previous population-based study [5]. First, we required at least two redeemed prescriptions within the same medication class, representing a confirmation of the mental disorder by the treating physician. Second, we excluded prescriptions for anxiolytics, which may be used for transient stress/adjustment reactions or insomnia, and quetiapine, which may be prescribed off-label for non-psychiatric conditions [27, 28]. Third, to account for the potential off-label use of antidepressants and antipsychotics for somatic conditions such as chronic pain, migraines, urinary incontinence, irritable bowel syndrome, and dementia, individuals diagnosed with any of these somatic conditions required a hospital admission for mental disorders to meet our outcome of any mental disorder. 2) We then reran our analysis among individuals without preexisting physical conditions (CCI = 0) to account for a potential impact of the cumulative burden of more than one chronic condition besides cancer. 3) Lastly, recognizing the well-established risk factor of preexisting mental disorders, we changed exclusion criteria, so we could also include cancer patients and comparisons showing a history of mental disorders prior to their cancer diagnosis or index date. All analyses were conducted using R, version 4.4.1 [29].

## Results

We included a total of 1,320,448 individuals in our study, consisting of 289,391 cancer patients and 1,031,057 matched population-comparisons (Table 1). Median follow-up was 14.9 years in the cancer and 16.1 years in the comparison cohort. Overall, 79,131 individuals (33%) in the cancer and 443,963 (43%) in the comparison cohort were censored due to the end of study period or emigration. Competing events of death occurred in 94,142 individuals (33%) in the cancer and 160,915 (16%) in the comparison cohort.

**Table 1:** Baseline characteristics for cancer patients and comparisons (*N* = 1,320,448)

No. (%)	Cancer	Comparison
**No. of individuals**	289,391	1,031,057
**Sex**		
Women	136,921 (47%)	461,521 (45%)
Men	152,470 (53%)	569,536 (55%)
**Age**, median (IQR), years	66 (56, 74)	65 (54, 73)
**Socioeconomic position**		
Employed or student	104,940 (36%)	419,291 (41%)
Retired	161,577 (56%)	548,949 (53%)
Unemployed	4660 (2%)	15,461 (2%)
Disabled or other	8046 (3%)	29,650 (3%)
Missing	10,168 (4%)	17,706 (2%)
**Cohabitation status**		
Married	185,478 (64%)	672,308 (65%)
Widowed	41,860 (14%)	136,362 (13%)
Not married	31,588 (11%)	120,378 (12%)
Divorced	30,465 (11%)	102,009 (10%)
**Charlson Comorbidity Index (CCI)**		
0	242,239 (84%)	901,228 (87%)
1	34,747 (12%)	99,834 (10%)
2	8848 (3%)	22,478 (2%)
≥3	3557 (1%)	7517 (1%)
**Cancer type**		
Breast	49,373 (17%)	
Prostate	42,800 (15%)	
Lung	25,586 (9%)	
Colon	25,433 (9%)	
Malignant melanoma	20,747 (7%)	
Rectum	16,494 (6%)	
Bladder, urinary tract	9672 (3%)	
Leukemia	8259 (3%)	
Oropharynx, oral cavity	7913 (3%)	
Endometrium	7868 (3%)	
Non-Hodgkin lymphoma	6154 (2%)	
Kidney	5949 (2%)	
Ovary	5812 (2%)	
Cervix, uteri	5203 (2%)	
Testis	4916 (2%)	
Pancreas	4720 (2%)	
Stomach	4650 (2%)	
Brain, nervous system	4248 (2%)	
Multiple myeloma	3593 (1%)	
Sarcoma	3539 (1%)	
Esophagus	2968 (1%)	
Liver	2708 (0.9%)	
Larynx	2628 (0.9%)	
Thyroid	2526 (0.9%)	
Vulva, vagina	1126 (0.4%)	
Anal	1088 (0.4%)	
Hodgkin lymphoma	900 (0.3%)	
Small intestine	815 (0.3%)	
Other	11,703 (4%)	

*IQR* Interquartile range, *CCI* Charlson Comorbidity Index.

Across all cancer types combined, 116,118 patients were identified with any incident mental disorder requiring an inpatient/outpatient hospital admission or a psychotropic medication, corresponding to an IR per 1000 person-years of 59.9 [95%-CI: 59.5–60.2]. Of these, 22,923 had a hospital admission and 109,781 redeemed a psychotropic medication. The HR of any mental disorder in cancer patients was 2.3 [95%-CI: 2.3–2.3] when compared to matched population-comparisons.

**Fig. 1:**
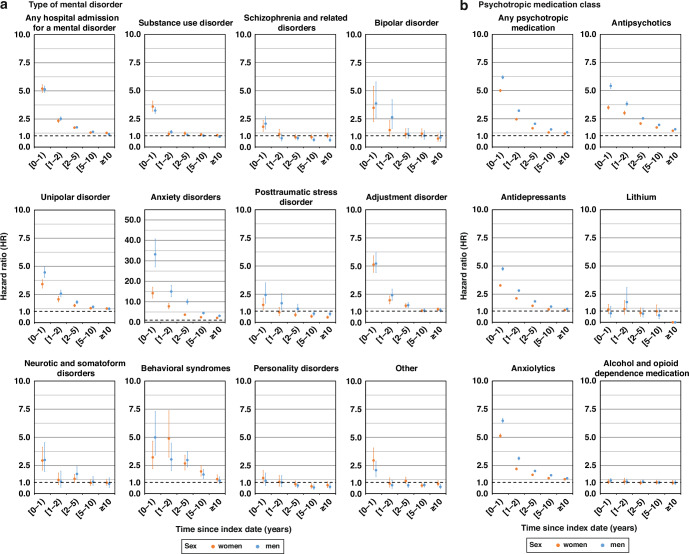
Time course of hazard ratios (HR) with 95% confidence intervals (CI) for mental disorders in cancer patients. HR are displayed across time for men and women, stratified by **a** type of mental disorder and **b** psychotropic medication class.

The IR of any mental disorder was highest in the first year after cancer diagnosis (167.0 [95%-CI: 165.4-168.6]) and decreased rapidly thereafter (Table 2). The same time course also applied for the HR of any mental disorder, with a 12.3-fold [95%-CI: 11.9–12.7] increased risk in the first year, as well as for HRs of different types of mental disorders and psychotropic medications (Fig. 1, Supplement S3). Highest IRs and HRs were observed for anxiety disorders, unipolar depression, substance use disorders, as well as for anxiolytics and antidepressants (Table 3). Men with cancer exhibited a particularly high IR compared to women for substance use disorders (men: 3.6 [95%-CI: 3.4–3.7]; women: 1.9 [95%-CI: 1.8–1.9]), and a higher HR of anxiety disorders (men: 8.5 [95%-CI: 8.0–9.0]; women: 3.6 [95%-CI: 3.5–3.8]). IRs and HRs for any mental disorder varied across cancer types, being highest in patients with pancreatic, lung, esophageal, and brain and nervous system cancers (Table 4, Supplement S4).

**Table 2:** Time course of the incidence rate (IR) and hazard ratio (HR) for mental disorders in cancer patients, stratified by outcome assessment

	Any mental disorder^1^		Hospital admission for a mental disorder		Psychotropic medication prescription	
	IR^2^(95%-CI)	HR^3^(95%-CI)	IR(95%-CI)	HR(95%-CI)	IR(95%-CI)	HR(95%-CI)
**0–1 year**	167.0 (165.4; 168.6)	12.3 (11.9; 12.7)	20.8 (20.3; 21.4)	5.2 (4.9; 5.4)	154.9 (153.4; 156.4)	5.5 (5.5; 5.6)
**1–2 years**	38.8 (38.2; 39.4)	7.4 (7.2; 7.6)	5.9 (5.7; 6.1)	2.4 (2.3; 2.5)	36.6 (36.0; 37.2)	2.8 (2.8; 2.9)
**2–5 years**	28.5 (28.2; 28.9)	3.4 (3.4; 3.5)	5.3 (5.1; 5.4)	1.7 (1.7; 1.8)	26.6 (26.3; 27.0)	1.9 (1.8; 1.9)
**5–10 years**	16.0 (15.8; 16.3)	1.8 (1.8; 1.8)	3.3 (3.3; 3.4)	1.3 (1.3; 1.4)	15.0 (14.8; 15.2)	1.4 (1.4; 1.4)
**≥ 10 years**	10.9 (10.7; 11.1)	1.4 (1.4; 1.4)	2.6 (2.5; 2.7)	1.2 (1.2; 1.3)	10.2 (10.0; 10.4)	1.2 (1.2; 1.3)

^1^ Any mental disorder requiring hospital admission and/or psychotropic medication prescription.

^2^ Incidence rate (IR) per 1000 person years in the cancer cohort.

^3^ Hazard ratio (HR) in the cancer cohort versus comparison cohort.

**Table 3:** Incidence rates (IR) and hazard ratios (HR) for mental disorders in cancer patients, stratified by sex, type of mental disorder and psychotropic medication class (*N* = 1,320,460)

	Sex	*N*_Cancer_	*N*_Comparison_	IR^2^ (95%-CI)	HR^3^ (95%-CI)
**Any mental disorder**^**1**^	Total	116,118	289,704	59.9 (59.5; 60.2)	2.3 (2.3; 2.3)
	Men	59,013	145,971	63.4 (62.9; 63.9)	2.5 (2.5; 2.6)
	Women	57,105	143,733	56.6 (56.2; 57.1)	2.0 (2.0; 2.1)
**Hospital admission for a mental disorder**					
Any hospital admission for a mental disorder	Men	11,707	35,843	10.8 (10.6; 11.0)	1.9 (1.9; 2.0)
	Women	11,216	30,752	8.8 (8.6; 9.0)	1.7 (1.7; 1.8)
Substance use disorder	Men	3938	17,083	3.6 (3.4; 3.7)	1.3 (1.3; 1.4)
	Women	2434	8462	1.9 (1.8; 1.9)	1.3 (1.2; 1.4)
Schizophrenia and related disorders	Men	313	2100	0.3 (0.3; 0.3)	0.9 (0.8; 1.0)
	Women	426	1980	0.3 (0.3; 0.4)	1.1 (1.0; 1.2)
Bipolar disorder	Men	160	626	0.1 (0.1; 0.2)	1.5 (1.3; 1.8)
	Women	186	713	0.1 (0.1; 0.2)	1.3 (1.1; 1.5)
Unipolar depression	Men	3769	12,327	3.4 (3.3; 3.5)	1.9 (1.8; 1.9)
	Women	4186	13,602	3.2 (3.1; 3.3)	1.6 (1.5; 1.6)
Anxiety disorders	Men	3213	2203	2.9 (2.8; 3.0)	8.5 (8.0; 9.0)
	Women	3202	3988	2.4 (2.3; 2.5)	3.6 (3.5; 3.8)
Posttraumatic stress disorder	Men	253	1292	0.2 (0.2; 0.3)	1.0 (0.9; 1.2)
	Women	335	1719	0.3 (0.2; 0.3)	0.6 (0.6; 0.7)
Adjustment disorder	Men	1187	4129	1.1 (1.0; 1.1)	1.7 (1.6; 1.8)
	Women	1946	5614	1.5 (1.4; 1.6)	1.6 (1.5; 1.7)
Neurotic and somatoform disorders	Men	162	627	0.1 (0.1; 0.2)	1.4 (1.2; 1.7)
	Women	310	1166	0.2 (0.2; 0.3)	1.3 (1.1; 1.4)
Behavioral syndromes	Men	390	1091	0.4 (0.3; 0.4)	2.0 (1.8; 2.3)
	Women	421	869	0.3 (0.3; 0.4)	2.2 (2.0; 2.5)
Personality disorders	Men	142	1054	0.1 (0.1; 0.2)	0.7 (0.6; 0.8)
	Women	206	1132	0.2 (0.1; 0.2)	0.8 (0.7; 1.0)
Other	Men	246	1525	0.2 (0.2; 0.3)	0.9 (0.8; 1.0)
	Women	326	1352	0.3 (0.2; 0.3)	1.1 (1.0; 1.2)
**Psychotropic medication prescription**					
Any psychotropic medication prescription	Men	55,434	134,323	58.4 (58.0; 58.9)	2.6 (2.6; 2.6)
	Women	54,347	136,945	53.0 (52.5; 53.4)	2.0 (2.0; 2.1)
Antipsychotics	Men	18,491	39,325	16.7 (16.5; 17.0)	2.9 (2.8; 2.9)
	Women	15,453	32,456	11.9 (11.7; 12.1)	2.3 (2.3; 2.4)
Antidepressants	Men	35,260	95,636	35.1 (34.8; 35.5)	2.3 (2.2; 2.3)
	Women	35,253	101,365	31.3 (30.9; 31.6)	1.7 (1.7; 1.8)
Lithium	Men	69	434	0.1 (0.1; 0.1)	1.0 (0.7; 1.2)
	Women	119	553	0.1 (0.1; 0.1)	1.1 (0.9; 1.3)
Anxiolytics	Men	22,876	46,948	21.7 (21.4; 21.9)	3.0 (2.9; 3.0)
	Women	26,567	57,193	22.6 (22.4; 22.9)	2.4 (2.3; 2.4)
Alcohol and opioid dependence medication	Men	1008	5289	0.9 (0.8; 1.0)	1.1 (1.0; 1.2)
	Women	663	3089	0.5 (0.5; 0.5)	1.0 (1.0; 1.1)

^1^ Any mental disorder requiring hospital admission and/or psychotropic medication prescription.

^2^ Incidence rate (IR) per 1000 person years in the cancer cohort.

^3^ Hazard ratio (HR) in the cancer cohort versus comparison cohort.

**Table 4:** Cancer entity specific incidence rates (IR) and hazard ratios (HR) for any mental disorder

Cancer entity	*N*_Cancer_	*N*_Comparison_	IR^1^ (95%-CI)	HR^2^ (95%-CI)
Breast	20,659	50,830	46.9 (46.3; 47.6)	1.8 (1.8; 1.8)
Prostate	16,439	40,024	55.3 (54.5; 56.2)	2.0 (2.0; 2.0)
Lung	12,050	25,263	230.9 (226.8; 235.0)	8.3 (8.1; 8.6)
Colon	10,178	25,950	63.6 (62.4; 64.9)	2.0 (2.0; 2.1)
Malignant melanoma	6129	19,171	28.1 (27.4; 28.8)	1.3 (1.2; 1.3)
Rectum	6928	17,168	66.3 (64.8; 67.9)	2.3 (2.2; 2.4)
Bladder, urinary tract	3847	10,600	74.2 (71.9; 76.6)	2.4 (2.3; 2.5)
Leukemia	2619	8359	47.8 (46.0; 49.7)	1.8 (1.7; 1.9)
Oropharynx, oral cavity	3678	8103	76.0 (73.6; 78.5)	2.9 (2.8; 3.1)
Endometrium	3165	8607	43.5 (42.0; 45.0)	1.5 (1.4; 1.5)
Non-Hodgkin lymphoma	2007	4524	46.1 (44.1; 48.1)	2.0 (1.9; 2.1)
Kidney	2470	5507	67.9 (65.2; 70.6)	2.8 (2.6; 2.9)
Ovary	2725	6645	88.3 (85.0; 91.7)	3.3 (3.1; 3.5)
Cervix, uteri	2169	6245	43.7 (41.9; 45.5)	1.8 (1.7; 1.9)
Testis	1351	4693	21.1 (20.0; 22.2)	1.3 (1.2; 1.4)
Pancreas	1972	4300	303.4 (290.2; 317.0)	11.9 (11.0; 12.9)
Stomach	1907	4797	139.2 (133.1; 145.6)	4.9 (4.5; 5.2)
Brain, nervous system	2166	4170	192.0 (184.1; 200.2)	10.3 (9.4; 11.1)
Multiple myeloma	1471	3629	98.7 (93.7; 103.8)	3.6 (3.4; 3.9)
Sarcoma	1644	3399	75.4 (71.9; 79.2)	3.3 (3.1; 3.5)
Esophagus	1277	2749	225.9 (213.7; 238.45)	8.8 (8.1; 9.67)
Liver	975	2453	187.6 (176.1; 199.7)	7.0 (6.3; 7.7)
Larynx	1207	2903	72.7 (68.7; 76.9)	2.7 (2.5; 2.9)
Thyroid	796	2396	30.2 (28.1; 32.3)	1.4 (1.3; 1.5)
Vulva, vagina	497	1294	64.5 (59.0; 70.3)	1.9 (1.7; 2.1)
Anal	497	1076	68.2 (62.4; 74.4)	2.6 (2.4; 3.0)
Hodgkin lymphoma	263	647	33.9 (30.0; 38.2)	1.9 (1.6; 2.2)
Small intestine	340	683	71.8 (64.4; 79.7)	3.3 (2.8; 3.8)
Other	4692	13,519	71.4 (69.4; 73.5)	2.7 (2.6; 2.8)

^1^ Incidence rate (IR) per 1000 person years.

^2^ Hazard ratio (HR) in the cancer cohort versus comparison cohort.

**Fig. 2:**
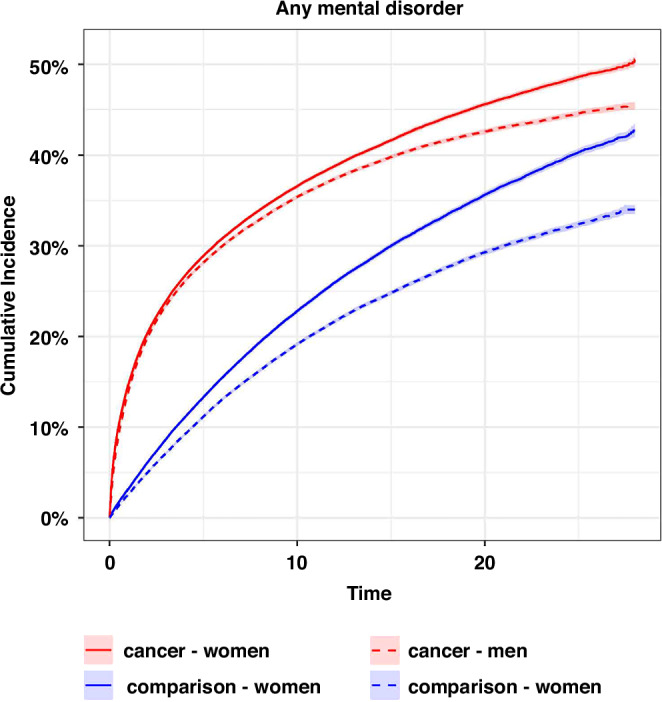
Cumulative incidence for a mental disorder following a cancer diagnosis. Cumulative incidence (%) for men and women with cancer, as well as comparisons, across 25 years.

The cumulative incidence for any mental disorder was estimated for the entire cancer cohort and revealed a 1-year risk of 14% in men and 15% in women with cancer, in contrast to 3% in both sexes for cancer-free comparisons. This risk increased to 45% in men and 49% in women with cancer 25-years following the cancer diagnosis, in contrast to 33% and 39% in cancer-free men and women (Fig. 2). Cumulative incidence rates by cancer type are presented in the Supplement (Fig S1). The overall patterns are comparable across cancer types, although the slopes vary depending on the specific cancer type.

### Sensitivity analyses

Across all sensitivity analyses, the overall pattern of results remained unchanged regarding the temporal course and most prevalent types of mental disorders. Although the IR of any mental disorder in cancer patients varied depending on the definition of outcome and population of interest, the HR of any mental disorder consistently ranged between 2.0 and 2.3, indicating a two-fold increased risk for cancer patients in relation to the comparison cohort.

When applying stricter outcome criteria for psychotropic medication (Supplement S5), requiring at least two prescriptions within the same medication class, the IR of any mental disorder decreased to 38.8 [95%-CI: 38.5–39.0] compared to 59.9 in the main analysis. The HR decreased slightly from 2.3 in the main analysis to 2.0 [95%-CI: 2.0–2.1]. The exclusion of anxiolytics and quetiapine reduced the IR to 46.4 [95%-CI: 46.1–46.7] and the HR to 2.1 [95%-CI: 2.1–2.2]. The exclusion of potential off-label use of antidepressants and antipsychotics for somatic conditions reduced the IR to 54.1 [95%-CI: 53.8–54.4] and the HR remained at 2.3 [95%-CI: 2.2–2.3]. Addressing further influencing factors, we reran our analysis excluding individuals with preexisting physical conditions (Supplement S6), resulting in minor changes compared to the main analysis, with an IR of 56.1 [95%-CI: 55.8–56.5], and a HR of 2.3 [95%-CI: 2.3–2.3]. Lastly, we reran the analysis additionally including patients and comparisons with preexisting mental disorders, which is particularly relevant for clinical healthcare implications (Supplement S7) – almost doubling the sample size to 445,124 cancer patients and 2,163,890 comparisons – leading to an elevated IR of 97.9 [95%-CI: 97.5–98.3], whereby women with cancer (IR 100.6 [95%-CI: 100.1–101.2]) having slightly higher IR than men with cancer (IR 94.8 [95%-CI: 94.2–95.4]). The HR comparing the risk in cancer patients to comparisons without cancer, however, again remained relatively stable at 2.1 [95%-CI: 2.1–2.1].

## Discussion

This study represents one of the largest studies to date investigating the incidence of psychiatric diagnoses and prescription of psychotropics among cancer patients, providing a comprehensive and fine-grained stratification across the full spectrum of types of mental disorders and tumor types, based on a nearly three-decade follow-up. We estimated IRs of mental disorders and HRs in relation to cancer-free individuals in a nationwide population-based cohort of newly diagnosed cancer patients. Most important, cancer patients exhibit a two-fold increased risk of developing any incident mental disorder compared to cancer-free individuals, being highest in the first year following the cancer diagnosis but remaining elevated for over ten years. One in two cancer patients developed an incident mental disorder within 25 years following their cancer diagnosis. The most common disorders were anxiety, depression and substance use disorders. Men with cancer were particularly at risk for developing anxiety and substance use disorders.

Our findings, together with extensive previous research [6, 7, 30], indicate that anxiety disorders and depression are the most common psychiatric consequences of a cancer diagnosis, which likely arise from the inherent uncertainty and multifaceted challenges that cancer and its treatment impose. Our results further suggest that certain mental disorders, such as personality disorders or schizophrenia, do not exhibit an increased risk relative to the general population. This is plausible, given that these conditions typically manifest earlier in life and are generally not triggered by a single critical event [31, 32]. Notably, we demonstrate that the risk of developing posttraumatic stress disorder after cancer is not elevated, contributing to the ongoing debate about whether cancer itself may be traumatic, as well as low rates of cancer-related posttraumatic stress disorder [33]. In contrast to Lu et al. (2016) [10], who reported an increased risk of stress reaction/adjustment disorder among cancer patients, we analyzed posttraumatic stress disorder and adjustment disorder separately. This methodological distinction may explain the differing results.

To a varying extent, the temporal pattern of results was similar across most common types of mental disorders. The increase in risk immediately after cancer diagnosis, followed by a gradually declining, yet elevated risk over time, is consistent with previous research [7–10, 12–14, 34] that has focused primarily on depression.

This study is among the first to demonstrate sex differences in the incidence and risk of mental disorders using population-based psychiatric outcome data. Internalizing mental disorders, such as depression, anxiety and stress-related disorders are more commonly diagnosed in women [35]. In contrast, our findings reveal slightly higher IRs for depression and anxiety disorders among men with cancer. Notably, the risk of anxiety disorders in men with cancer was most strongly elevated, showing an 8.5-fold increase compared to men without cancer. This might be explained by gender-specific coping-strategies and societal expectations in dealing with cancer: women are more likely to seek social support and employ emotional coping-mechanisms, whereas men may tend to show avoidance – possible maintaining psychological distress – and perceive having cancer as a weakness [36]. Externalizing mental disorders, particularly substance use disorders, are typically more prevalent in men [35], a pattern that aligns with our observations in the oncological population. We extend previous results by demonstrating that both sexes face an increased risk for substance use disorder compared to cancer-free individuals.

Our findings indicate highest rates of mental disorders in cancers with poor prognosis. Studies comparing mental disorder rates across all tumor types are limited [7, 10, 37], and our findings are the first to comprehensively stratify results across common, but also rare types of cancers. Psychological distress associated with a poor prognosis and feelings of uncertainty about the disease trajectory may elicit anxiety and mood disturbances. Our findings highlight the need for proactive mental health strategies in oncology care. In addition to tailored interventions for high-risk groups, population-based approaches such as routine screening for psychiatric symptoms, especially within the first year after diagnosis, and psychoeducational efforts for patients and clinicians to increase awareness and reduce stigma could promote early recognition and treatment of mental disorders. Supervision or evaluation by specialists in psychiatry or psychiatric nurses should optimally be an integrated part of diagnosing and treatment of cancer. Future studies should focus on vulnerable groups, identified in our analyses by tumor type, sex, and time since diagnosis.

Several sensitivity analyses strengthened the robustness of our findings. Of particular interest was the analysis that included patients and comparisons with preexisting mental disorders. Notably, the previously observed twofold increase in risk for developing any incident mental disorder among cancer patients remained unchanged, highlighting that the elevated vulnerability is independent of prior psychiatric history. However, the sex-specific patterns differed from the main analysis. While the main analysis showed higher incidence rates in men, this sensitivity analysis revealed slightly higher rates in women, aligning with patterns observed in the general population, thus potentially reflecting more real-world conditions. The main strength of the current study arises from the large population-based sample. By integrating data from multiple registries, we capture severe cases of mental disorders by admissions to psychiatric facilities, and milder cases managed through psychotropic medication prescriptions. This comprehensive approach minimizes biases related to population-selection and reliance on self-reported symptoms. The similar temporal pattern observed in both outcome measures suggests that cancer may affect both, severe and milder forms of mental disorders. Denmark’s healthcare system offers free and equal access to healthcare, which minimizes confounding by economic disparities in health insurance coverage or access to care. Finally, we contribute to the field by providing data with a nearly three-decade follow-up, offering incidence rates and risks across common and rare tumor types, which are often underrepresented due to small sample sizes, and by examining the full spectrum of mental disorders over time, providing insights that may inform clinical oncology practice.

Some limitations warrant discussion. First, mental disorders are under-detected and undertreated in primary care [38], and our data might not include patients who seek help outside the conventional medical system, or those who do not seek help at all [39]. Although mental health services in Denmark are largely tax-funded, inequities in access may persist, both in the geographical distribution of specialized services and in social or socioeconomic disparities that influence treatment utilization. We may thus underestimate the incidence and the healthcare burden of mental disorders among patients with cancer. One cannot exclude that some misclassifications between cancer and psychiatric disorders may have biased our results, such as an increased risk of brain cancer in the first year following hospitalization for depression [40]. However, we consider this bias to have minimal impact on the overall findings. We recognize that using registry data on psychotropic prescriptions as a proxy for mental disorders can be contentious, as it might be prescribed for other disorders of psychiatric symptoms rather than mental disorders. To mitigate potential overestimation, we conducted several sensitivity analyses as in our prior population-based study[5], excluding psychotropic medication that is often prescribed for non-psychiatric disorder, all demonstrating robust results. Lastly, we excluded individuals with preexisting mental disorders from our main analysis, representing a major predictor for the development of subsequent mental health issues. This approach was necessary in order to validly picture the downstream impact of cancer itself on incident mental disorders, yet, it limits the generalizability of the findings. We addressed this limitation by including them in a sensitivity analysis, and as expected, the sample size and cases with mental disorders increased drastically. Nevertheless, the increased relative difference between the cancer and comparison cohort remained stable.

## Conclusion

Cancer poses an increased risk of developing new-onset mental disorders, particularly depression and anxiety disorders. Although the first year following the cancer diagnosis represents a particularly vulnerable period, the risk of mental disorders remains elevated long after the initial cancer diagnosis. These results underscore the need for tailored psychiatric and psychotherapeutic interventions in oncology settings.

## Supplementary information

Springer et al_Incidence mental disorders following cancer_Supplement

## Data Availability

The data and code of this study is not available as it is property of Statistic Denmark and the Danish Health Data Authority. The data are available from the authorities, but restrictions apply.
