# Supplementary material for: Incident mental disorders following cancer: analysis of real-world psychiatric outcomes in a nationwide population-based cohort study in Denmark across three decades
Source: Br J Cancer. 2026 Apr 29;135(3):445–52. doi: 10.1038/s41416-026-03460-8 (PMC13372743; doi:10.1038/s41416-026-03460-8)
Supplement: Supplementary file 1 — Springer et al_Incidence mental disorders following cancer_Supplement [file 41416_2026_3460_MOESM1_ESM.docx]

**SUPPLEMENT**

**Table S1.** Inclusion and exclusion of mental disorders and psychotropic medication ………………...2

**Table S2.** Cancer diagnostic groups ……………………………………………………………………3

**Table S3.** Time course of the hazard ratios (HR) for mental disorders in cancer patients, stratified by type of mental disorder and psychotropic medication class (N = 1,320,448) ………………………….4

**Table S4.** Incidence rates (IR) and hazard ratios (HR) of registered hospital admissions for mental disorders and psychotropic medication prescriptions, stratified by cancer type (N = 1,320,448) ……...5

**Table S5.** Sensitivity analysis with stricter outcome criteria for psychotropic medication (N = 1,320,448) ………………………………………………………………………………………………7

**Table S6.** Sensitivity analysis excluding patients with cancer and comparisons with physical comorbidities (N = 1,121,781) ………………………………………………………………………….8

**Table S7.** Sensitivity analysis including patients with cancer and comparisons with preexisting mental disorders (N = 2,609,014) ………………………………………………………………………………9

**Figure S1**. Cumulative incidence for any mental disorder, stratified by cancer type………………....10

| **Table S1.** Inclusion and exclusion of mental disorders and psychotropic medication | |
| --- | --- |
| **Mental disorders** | **ICD-10 code** |
| **Inclusion** |  |
| Substance use disorder | F10-19 |
| Schizophrenia and related disorders | F20-29 |
| Bipolar disorder | F30-31, F34.0, F38.0 |
| Unipolar depression | F32-33 F34.1, F38.1 |
| Anxiety disorders | F40, F41 |
| Posttraumatic stress disorder | F43.0, F43.1 |
| Adjustment disorder | F43.2, F43.8, F43.9 |
| Neurotic and somatoform disorders | F42, F44, F45, F48 |
| Behavioral syndromes associated with  physiological disturbances and physical factors | F50-59 |
| Personality disorders | F60-69 |
| Other psychiatric disorders | F70-79, F99 |
| **Exclusion** |  |
| Dementia, delirium and other organic related mental  disorders | F00-F09 |
| Developmental disorders | F80-98 |
| **Psychotropic medication** | **ATC code** |
| **Inclusion** |  |
| Antipsychotics | N05A minus N05AN |
| Antidepressants | N06A |
| Lithium | N05AN |
| Anxiolytics | N05B excluding benzodiazepine derivatives (N05BA0: diazepam) |
| Medication for alcohol and opioid dependence | N07BB-N07BB04 |
| **Exclusion** |  |
| Hypnotics for sleep | N05C |
| Medication for dementia | N06D |
| Parasympathomimetics | N07A |
| Medication for smoking cessation | N07BA |
| Medication for Attention Deficit Hyperactivity Disorder | C02AC02, N06BA02, N06BA04, N06BA09 and N06BA12 |

| **Table S2.** Cancer diagnostic groups | |
| --- | --- |
| **Cancer site** | **ICD-10 code** |
| Oropharynx and oral cavity | C00, C01, C02, C03, C04, C05, C06, C07, C08, C09, C10, C11, C12, C13, C14, C30, C31 |
| Esophagus | C15 |
| Stomach | C16 |
| Small intestine | C17 |
| Colon | C18 |
| Rectum | C19, C20 |
| Anal | C21 |
| Liver | C22, C23, C24 |
| Pancreas | C25 |
| Larynx | C32 |
| Lung | C33, C34 |
| Sarcoma | C40, C41, C45-C49 |
| Malignant melanoma | C43 |
| Breast | C50 |
| Vulva and Vagina | C51, C52 |
| Cervix uteri | C53 |
| Endometrium | C54, C55 |
| Ovary | C56, C57.0-4 |
| Prostate | C61 |
| Testis | C62 |
| Kidney | C64 |
| Bladder and urinary tract | C65, C66, C67, C68, D09, D41.4 |
| Brain and nervous system | C70, C71, C72, D32, D33, D42, D43 |
| Thyroid | C73 |
| Hodgkin lymphoma (HL) | C81 |
| Non-Hodgkin lymphoma (NHL) | C82, C83, C84, C85, C96 |
| Multiple myeloma | C90 |
| Leukemia | C91, C92, C93, C94, C95 |
| Other | C26, C37, C38, C39, C58, C60, C63, C69, C74, C75, C76-C80, C86, C88 |

| **Table S3.** Time course of the hazard ratios (HR) for mental disorders in cancer patients, stratified by type of mental disorder and psychotropic medication class (N = 1,320,448) | | | | | | |
| --- | --- | --- | --- | --- | --- | --- |
| **HR (95%-CI)** | **Sex** | **0-1 year** | **1-2 years** | **2-5 years** | **5-10 years** | **≥10 years** |
| **Hospital admission** | | | | | |  |
| Any hospital admission for a mental disorder | Men | 5.1 (4.9; 5.4) | 2.5 (2.4; 2.7) | 1.8 (1.7; 1.8) | 1.4 (1.3; 1.4) | 1.1 (1.1; 1.2) |
|  | Women | 5.2 (4.9; 5.6) | 2.3 (2.2; 2.5) | 1.7 (1.7; 1.8) | 1.3 (1.2; 1.3) | 1.3 (1.2; 1.3) |
| Substance use disorder | Men | 3.2 (3.0; 3.5) | 1.3 (1.2; 1.5) | 1.1 (1.0; 1.2) | 1.0 (0.9; 1.1) | 0.9 (0.8; 1.0) |
|  | Women | 3.6 (3.2; 4.1) | 1.2 (1.0; 1.4) | 1.2 (1.1; 1.4) | 1.1 (1.0; 1.2) | 1.0 (1.0; 1.1) |
| Schizophrenia and related disorders | Men | 2.1 (1.6; 2.7) | 0.8 (0.5; 1.1) | 0.8 (0.6; 1.0) | 0.6 (0.5; 0.8) | 0.6 (0.5; 0.8) |
|  | Women | 1.8 (1.4; 2.4) | 1.1 (0.8; 1.6) | 0.9 (0.7; 1.1) | 0.9 (0.7; 1.1) | 1.0 (0.8; 1.2) |
| Bipolar disorder | Men | 3.9 (2.6; 5.8) | 2.6 (1.7; 4.2) | 1.1 (0.8; 1.6) | 1.0 (0.7; 1.5) | 0.8 (0.5; 1.4) |
|  | Women | 3.5 (2.2; 5.4) | 1.5 (1.0; 2.4) | 1.1 (0.8; 1.6) | 1.2 (0.9; 1.6) | 0.8 (0.5; 1.1) |
| Unipolar depression | Men | 4.5 (4.0; 5.0) | 2.6 (2.3; 2.9) | 1.8 (1.7; 2.0) | 1.4 (1.3; 1.5) | 1.2 (1.1; 1.3) |
|  | Women | 3.4 (3.1; 3.8) | 2.1 (1.8; 2.3) | 1.5 (1.4; 1.7) | 1.3 (1.2; 1.4) | 1.3 (1.2; 1.3) |
| Anxiety disorders | Men | 33.2 (27.0; 40.7) | 15.0 (12.5; 18.0) | 10.1 (8.8; 11.3) | 4.5 (4.0; 5.0) | 3.1 (2.7; 3.5) |
|  | Women | 14.2 (11.7; 17.1) | 7.8 (6.5; 9.3) | 3.7 (3.3; 4.1) | 2.4 (2.2; 2.7) | 2.1 (1.9; 2.3) |
| Posttraumatic stress disorder | Men | 2.4 (1.7; 3.5) | 1.7 (1.1; 2.6) | 1.2 (0.9; 1.6) | 0.8 (0.6; 1.0) | 0.8 (0.6; 1.0) |
|  | Women | 1.6 (1.1; 2.2) | 0.9 (0.6; 1.3) | 0.7 (0.5; 0.9) | 0.5 (0.4; 0.7) | 0.5 (0.4; 0.6) |
| Adjustment disorder | Men | 5.2 (4.4; 6.2) | 2.4 (2.0; 3.0) | 1.5 (1.3; 1.8) | 1.1 (0.9; 1.2) | 1.1 (0.9; 1.2) |
|  | Women | 5.1 (4.4; 5.9) | 2.0 (1.6; 2.3) | 1.5 (1.3; 1.7) | 1.0 (0.9; 1.2) | 1.2 (1.0; 1.3) |
| Neurotic and somatoform disorders | Men | 3.0 (2.0; 4.6) | 1.1 (0.6; 2.0) | 1.8 (1.3; 2.4) | 1.1 (0.7; 1.5) | 0.9 (0.6; 1.4) |
|  | Women | 2.9 (2.1; 4.1) | 1.2 (0.8; 1.8) | 1.3 (1.0; 1.7) | 1.0 (0.7; 1.2) | 1.0 (0.7; 1.3) |
| Behavioral syndromes | Men | 5.0 (3.4; 7.3) | 3.0 (2.1; 4.5) | 3.0 (2.4; 3.8) | 1.7 (1.4; 2.2) | 1.2 (0.9; 1.5) |
|  | Women | 3.2 (2.2; 4.7) | 4.9 (3.2; 7.4) | 2.7 (2.1; 3.4) | 2.0 (1.6; 2.5) | 1.3 (1.0; 1.7) |
| Personality disorders | Men | 1.2 (0.7; 1.9) | 1.0 (0.6; 1.6) | 0.7 (0.5; 1.0) | 0.6 (0.4; 0.8) | 0.6 (0.4; 0.9) |
|  | Women | 1.4 (0.9; 2.1) | 1.0 (0.7; 1.6) | 0.8 (0.6; 1.1) | 0.7 (0.5; 0.9) | 0.8 (0.5; 1.1) |
| Other | Men | 2.1 (1.5; 2.9) | 0.8 (0.5; 1.2) | 0.7 (0.6; 1.0) | 0.8 (0.6; 1.0) | 0.6 (0.4; 0.8) |
|  | Women | 3.0 (2.1; 4.1) | 0.9 (0.6; 1.4) | 1.1 (0.9; 1.5) | 0.7 (0.6; 0.9) | 0.9 (0.7; 1.1) |
| **Psychotropic medication** | | | | | |  |
| Any psychotropic medication | Men | 6.2 (6.0; 6.3) | 3.2 (3.1; 3.3) | 2.1 (2.0; 2.1) | 1.6 (1.5; 1.6) | 1.3 (1.3; 1.3) |
|  | Women | 5.0 (4.9; 5.1) | 2.5 (2.4; 2.5) | 1.7 (1.6; 1.7) | 1.3 (1.3; 1.3) | 1.2 (1.2; 1.2) |
| Antipsychotics | Men | 5.4 (5.2; 5.7) | 3.8 (3.6; 4.0) | 2.5 (2.4; 2.6) | 2.0 (1.9; 2.0) | 1.6 (1.5; 1.7) |
|  | Women | 3.5 (3.4; 3.7) | 3.0 (2.9; 3.2) | 2.1 (2.0; 2.2) | 1.7 (1.7; 1.8) | 1.4 (1.4; 1.5) |
| Antidepressants | Men | 4.8 (4.6; 4.9) | 2.8 (2.7; 2.9) | 1.9 (1.8; 1.9) | 1.4 (1.4; 1.4) | 1.2 (1.2; 1.2) |
|  | Women | 3.3 (3.2; 3.4) | 2.1 (2.1; 2.2) | 1.5 (1.4; 1.5) | 1.2 (1.1; 1.2) | 1.1 (1.1; 1.1) |
| Lithium | Men | 0.8 (0.5; 1.4) | 1.8 (1.0; 3.1) | 0.8 (0.5; 1.3) | 0.6 (0.3; 1.2) | NA |
|  | Women | 1.1 (0.8; 1.6) | 1.2 (0.7; 1.9) | 0.8 (0.5; 1.3) | 1.0 (0.6; 1.6) | NA |
| Anxiolytics | Men | 6.5 (6.3; 6.7) | 3.1 (3.0; 3.3) | 2.0 (2.0; 2.1) | 1.6 (1.6; 1.7) | 1.4 (1.3; 1.4) |
|  | Women | 5.1 (5.0; 5.3) | 2.2 (2.1; 2.3) | 1.7 (1.6; 1.7) | 1.4 (1.3; 1.4) | 1.3 (1.2; 1.3) |
| Alcohol and opioid dependence medication | Men | 1.2 (1.0; 1.4) | 1.1 (0.9; 1.3) | 1.1 (0.9; 1.2) | 1.0 (0.9; 1.1) | 1.0 (0.8; 1.2) |
|  | Women | 1.0 (0.9; 1.3) | 1.1 (0.8; 1.4) | 1.0 (0.8; 1.1) | 1.0 (0.8; 1.2) | 1.0 (0.8; 1.2) |
| Hazard ratio (HR) and 95% confidence interval (CI) in the cancer cohort versus comparison cohort | | | | | | |

| **Table S4.** Incidence rates (IR) and hazard ratios (HR) of registered hospital admissions for mental disorders and psychotropic medication prescriptions, stratified by cancer type (N = 1,320,448) | | | | |
| --- | --- | --- | --- | --- |
|  | **n _Cancer_** | **n _Comparison_** | **IR^1^ (95%-CI)** | **HR^2^ (95%-CI)** |
| **Any hospital admission for a mental disorder** | | | | |
| Breast | 4,307 | 10,733 | 7.7 (7.4; 7.9) | 1.7 (1.6; 1.7) |
| Prostate | 2,921 | 8,628 | 8.6 (8.3; 8.9) | 1.6 (1.5; 1.6) |
| Lung | 1,838 | 5,752 | 26.6 (25.4; 27.8) | 4.8 (4.5; 5.0) |
| Colon | 2,063 | 5,437 | 10.8 (10.3; 11.2) | 1.9 (1.8; 2.0) |
| Malignant melanoma | 1,446 | 5,020 | 5.8 (5.5; 6.1) | 1.2 (1.1; 1.2) |
| Rectum | 1,422 | 3,673 | 11.2 (10.6; 11.8) | 2.1 (1.9; 2.2) |
| Bladder, urinary tract | 665 | 2,277 | 10.7 (9.9; 11.5) | 1.8 (1.7; 2.0) |
| Leukemia | 474 | 1,987 | 7.3 (6.7; 8.0) | 1.3 (1.2; 1.4) |
| Oropharynx, oral cavity | 909 | 2,012 | 14.5 (13.6; 15.4) | 2.5 (2.3; 2.8) |
| Endometrium | 582 | 1,576 | 6.5 (6.0; 7.0) | 1.4 (1.3; 1.6) |
| Non-Hodgkin lymphoma | 410 | 1,053 | 7.9 (7.2; 8.7) | 1.6 (1.4; 1.8) |
| Kidney | 435 | 1,379 | 9.8 (8.9; 10.8) | 1.8 (1.6; 2.0) |
| Ovary | 467 | 1,378 | 11.7 (10.7; 12.8) | 2.4 (2.2; 2.7) |
| Cervix, uteri | 562 | 1,760 | 9.0 (8.3; 9.7) | 1.5 (1.4; 1.7) |
| Testis | 503 | 1,800 | 6.8 (6.2; 7.4) | 1.2 (1.1; 1.3) |
| Pancreas | 358 | 984 | 45.9 (41.3; 50.8) | 8.3 (7.3; 9.6) |
| Stomach | 381 | 1,121 | 22.1 (20.0; 24.4) | 3.7 (3.3; 4.3) |
| Brain, nervous system | 419 | 1,179 | 26.4 (23.9; 29.0) | 4.9 (4.3; 5.6) |
| Multiple myeloma | 258 | 818 | 13.3 (11.7; 15.0) | 2.4 (2.1; 2.8) |
| Sarcoma | 320 | 979 | 11.7 (10.4; 13.0) | 2.0 (1.7; 2.3) |
| Esophagus | 301 | 663 | 41.2 (36.7; 46.0) | 7.3 (6.3; 8.5) |
| Liver | 191 | 618 | 31.0 (26.8; 35.6) | 4.9 (4.1; 5.9) |
| Larynx | 293 | 762 | 13.9 (12.4; 15.5) | 2.2 (1.9; 2.5) |
| Thyroid | 212 | 746 | 6.7 (5.9; 7.7) | 1.2 (1.0; 1.4) |
| Vulva, vagina | 117 | 240 | 12.0 (9.9; 14.3) | 2.3 (1.8; 2.9) |
| Anal | 112 | 225 | 11.9 (9.8; 14.2) | 2.5 (2.0; 3.1) |
| Hodgkin lymphoma | 90 | 252 | 9.8 (7.9; 12.0) | 1.4 (1.1; 1.8) |
| Small intestine | 70 | 172 | 12.3 (9.6; 15.4) | 2.4 (1.8; 3.2) |
| Other | 797 | 3,371 | 9.6 (9.0; 10.3) | 1.7 (1.5; 1.8) |
| **Any psychotropic medication prescription** | | | | |
| Breast | 19,675 | 48,410 | 43.9 (43.3; 44.6) | 1.8 (1.8; 1.8) |
| Prostate | 15,699 | 37,175 | 52.2 (51.4; 53.1) | 2.1 (2.0; 2.1) |
| Lung | 11,580 | 23,609 | 218.4 (214.4; 222.4) | 8.6 (8.4; 8.8) |
| Colon | 9,548 | 24,571 | 58.6 (57.4; 59.7) | 2.0 (2.0; 2.1) |
| Malignant melanoma | 5,701 | 17,603 | 25.7 (25.1; 26.4) | 1.3 (1.2; 1.3) |
| Rectum | 6,495 | 16,197 | 60.7 (59.2; 62.2) | 2.2 (2.2; 2.3) |
| Bladder, urinary tract | 3,633 | 9,998 | 69.0 (66.7; 71.2) | 2.4 (2.3; 2.5) |
| Leukemia | 2,494 | 7,790 | 45.0 (43.2; 46.8) | 1.9 (1.8; 1.9) |
| Oropharynx, oral cavity | 3,417 | 7,463 | 68.9 (66.6; 71.2) | 3.0 (2.8; 3.1) |
| Endometrium | 3,027 | 8,311 | 41.2 (39.7; 42.6) | 1.5 (1.4; 1.5) |
| Non-Hodgkin lymphoma | 1,876 | 4,139 | 42.3 (40.4; 44.3) | 2.0 (1.9; 2.2) |
| Kidney | 2,351 | 5,069 | 63.5 (61.0; 66.1) | 2.9 (2.7; 3.0) |
| Ovary | 2,619 | 6,346 | 83.5 (80.3; 86.7) | 3.3 (3.2; 3.5) |
| Cervix, uteri | 1,984 | 5,845 | 38.7 (37.0; 40.4) | 1.8 (1.7; 1.9) |
| Testis | 1,200 | 4,135 | 18.3 (17.2; 19.3) | 1.3 (1.2; 1.4) |
| Pancreas | 1,837 | 4,025 | 275.3 (262.9; 288.1) | 11.9 (11.0; 13.0) |
| Stomach | 1,772 | 4,484 | 124.8 (119.1; 130.7) | 4.8 (4.4; 5.1) |
| Brain, nervous system | 2,059 | 3,814 | 178.2 (170.6; 186.0) | 10.8 (9.9; 11.7) |
| Multiple myeloma | 1,408 | 3,415 | 92.5 (87.8; 97.4) | 3.7 (3.4; 3.9) |
| Sarcoma | 1,564 | 3,067 | 70.3 (66.9; 7.9) | 3.5 (3.2; 3.7) |
| Esophagus | 1,152 | 2,546 | 190.6 (179.8; 201.8) | 8.2 (7.4; 9.0) |
| Liver | 894 | 2,290 | 169.3 (158.5; 180.7) | 6.9 (6.2; 7.6) |
| Larynx | 1,130 | 2,666 | 66.0 (62.3; 70.0) | 2.7 (2.5; 2.9) |
| Thyroid | 730 | 2,191 | 27.2 (25.2; 29.2) | 1.4 (1.3; 1.6) |
| Vulva, vagina | 452 | 1,252 | 56.5 (51.5; 61.9) | 1.8 (1.6; 2.0) |
| Anal | 453 | 1,006 | 59.8 (54.4; 65.4) | 2.5 (2.2; 2.8) |
| Hodgkin lymphoma | 241 | 554 | 30.4 (26.7; 34.4) | 2.0 (1.7; 2.4) |
| Small intestine | 319 | 622 | 65.8 (58.9; 73.3) | 3.4 (2.9; 3.9) |
| Other | 4,471 | 12,675 | 66.7 (64.8; 68.7) | 2.8 (2.7; 2.9) |
| ^1^ Incidence rate (IR) per 1,000 person years ^2^ Hazard ratio (HR) in the cancer cohort versus comparison cohort | | | | |

| **Table S5.** Sensitivity analysis with stricter outcome criteria for psychotropic medication (N = 1,320,448) | | | | | |
| --- | --- | --- | --- | --- | --- |
|  | **Sex** | **n _Cancer_** | **n _Comparison_** | **IR^2^ (95%-CI)** | **HR^3^ (95%-CI)** |
| **At least two prescriptions within the same drug class** | | | | | |
| **Any mental disorder**^1^ | Total | 81,370 | 219,563 | 38.8 (38.5; 39.0) | 2.0 (2.0; 2.1) |
|  | Men | 40,654 | 110,002 | 40.9 (40.5; 41.3) | 2.3 (2.2; 2.3) |
|  | Women | 40,716 | 109,561 | 36.8 (36.4; 37.1) | 1.9 (1.8; 1.9) |
| Any psychotropic medication | Men | 35,337 | 95,024 | 34.8 (34.5; 35.2) | 2.2 (2.2; 2.3) |
|  | Women | 36,556 | 100,533 | 32.4 (32.0; 32.7) | 1.8 (1.8; 1.8) |
| Antipsychotics | Men | 8,659 | 24,012 | 7.8 (7.6; 7.9) | 2.2 (2.2; 2.3) |
|  | Women | 7,644 | 20,710 | 5.8 (5.7; 6.0) | 1.8 (1.8; 1.9) |
| Antidepressants | Men | 24,822 | 73,514 | 23.9 (23.6; 24.2) | 2.1 (2.0; 2.1) |
|  | Women | 26,043 | 80,253 | 22.1 (21.8; 22.3) | 1.6 (1.6; 1.6) |
| Lithium | Men | 51 | 348 | 0.1 (0.0; 0.1) | 0.9 (0.7; 1.2) |
|  | Women | 102 | 433 | 0.1 (0.1; 0.1) | 1.2 (1.0; 1.5) |
| Anxiolytics | Men | 11,172 | 24,020 | 10.2 (10.0; 10.4) | 2.8 (2.8; 2.9) |
|  | Women | 14,262 | 31,137 | 11.4 (11.2; 11.6) | 2.3 (2.3; 2.4) |
| Alcohol and opioid dependence medication | Men | 546 | 2,968 | 0.5 (0.4; 0.5) | 1.1 (1.0; 1.2) |
|  | Women | 380 | 1,941 | 0.3 (0.2; 0.3) | 1.0 (0.9; 1.1) |
| **Exclusion of anxiolytics and quetiapine** | | | | | |
| **Any mental disorder**^1^ | Total | 95,840 | 247,553 | 46.4 (46.1; 46.7) | 2.1 (2.1; 2.2) |
|  | Men | 49,601 | 126,392 | 51.0 (50.6; 51.5) | 2.4 (2.4; 2.4) |
|  | Women | 46,239 | 121,161 | 42.3 (41.9; 42.7) | 1.9 (1.9; 1.9) |
| Any psychotropic medication | Men | 45,289 | 113,340 | 45.7 (45.3; 46.1) | 2.5 (2.4; 2.5) |
|  | Women | 42,712 | 113,168 | 38.3 (38.0; 38.7)­ | 1.9 (1.9; 1.9) |
| Antipsychotics | Men | 17,255 | 32,869 | 15.5 (15.3; 15.8) | 3.2 (3.2; 3.3) |
|  | Women | 14,279 | 27,452 | 11.0 (10.8; 11.2) | 2.6 (2.5; 2.6) |
| Antidepressants | Men | 35,260 | 95,636 | 35.1 (34.8; 35.5) | 2.3 (2.2; 2.3) |
|  | Women | 35,253 | 101,365 | 31.3 (30.9; 31.6) | 1.7 (1.7; 1.8) |
| Lithium | Men | 69 | 434 | 0.1 (0.1; 0.1) | 1.0 (0.7; 1.2) |
|  | Women | 119 | 553 | 0.1 (0.1; 0.1) | 1.1 (0.9; 1.3) |
| Alcohol and opioid dependence medication | Men | 1,008 | 5,289 | 0.9 (0.8; 1.0) | 1.1 (1.0; 1.2) |
|  | Women | 663 | 3,089 | 0.5 (0.5; 0.5) | 1.0 (1.0; 1.1) |
| **Exclusion of off-label use of antidepressants and antipsychotics for somatic conditions** | | | | | |
| **Any mental disorder**^1^ | Total | 106,043 | 265,899 | 54.1 (53.8; 54.4) | 2.3 (2.2; 2.3) |
|  | Men | 53,442 | 133,546 | 56.9 (56.4; 57.4) | 2.5 (2.5; 2.5) |
|  | Women | 52,601 | 132,353 | 51.5 (51.1; 52.0) | 2.1 (2.0; 2.1) |
| Any psychotropic medication | Men | 47,654 | 115,750 | 49.5 (49.1; 50.0) | 2.6 (2.5; 2.6) |
|  | Women | 48,132 | 120,997 | 46.1 (45.7; 46.5) | 2.0 (2.0; 2.1) |
| Antipsychotics | Men | 10,484 | 21,899 | 9.4 (9.2; 9.6) | 2.9 (2.9; 3.0) |
|  | Women | 8,762 | 18,444 | 6.7 (6.6; 6.9) | 2.4 (2.3; 2.4) |
| Antidepressants | Men | 27,599 | 75,513 | 27.0 (26.7; 27.3) | 2.2 (2.2; 2.3) |
|  | Women | 28,226 | 81,433 | 24.5 (24.2; 24.8) | 1.8 (1.7; 1.8) |
| Lithium | Men | 69 | 434 | 0.1 (0.1; 0.1) | 1.0 (0.7; 1.2) |
|  | Women | 119 | 553 | 0.1 (0.1; 0.1) | 1.1 (0.9; 1.3) |
| Anxiolytics | Men | 22,876 | 46,948 | 21.7 (21.4; 21.9) | 3.0 (2.9; 3.0) |
|  | Women | 26,567 | 57,193 | 22.6 (22.4; 22.9) | 2.4 (2.3; 2.4) |
| Alcohol and opioid dependence medication | Men | 1,008 | 5,289 | 0.9 (0.8; 1.0) | 1.1 (1.0; 1.2) |
|  | Women | 663 | 3,089 | 0.5 (0.5; 0.5) | 1.0 (1.0; 1.1) |
| Incidence rates (IR) and hazard ratios (HR) for any mental disorders and psychotropic medication classes  ^1^ Any mental disorder requiring a hospital admission and/or psychotropic medication  ^2^ Incidence rate (IR) per 1,000 person years in the cancer cohort  ^3^ Hazard ratio (HR) in the cancer cohort versus comparison cohort | | | | | |

| **Table S6.** Sensitivity analysis excluding patients with cancer and comparisons with physical comorbidities (N = 1,121,781) | | | | | |
| --- | --- | --- | --- | --- | --- |
|  | **Sex** | **n _Cancer_** | **n _Comparison_** | **IR^2^ (95% CI)** | **HR^3^ (95% CI)** |
| **Any mental disorder**^1^ | Total | 95,309 | 244,575 | 56.1 (55.8; 56.5) | 2.3 (2.3; 2.3) |
|  | Men | 46,038 | 117,030 | 58.7 (58.2; 59.3) | 2.6 (2.6; 2.6) |
|  | Women | 49,271 | 127,545 | 53.9 (53.4; 54.3) | 2.1 (2.0; 2.1) |
| **Hospital Admission** | | | | | |
| Any hospital admission for a mental disorder | Men | 9,090 | 29,534 | 9.9 (9.7; 10.2) | 1.9 (1.8; 1.9) |
|  | Women | 9,732 | 27,556 | 8.4 (8.3; 8.6) | 1.7 (1.7; 1.8) |
| Substance use disorder | Men | 3,064 | 14,169 | 3.3 (3.2; 3.4) | 1.3 (1.2; 1.3) |
|  | Women | 2,133 | 7,529 | 1.8 (1.7; 1.9) | 1.3 (1.3; 1.4) |
| Schizophrenia and related disorders | Men | 266 | 1,818 | 0.3 (0.3; 0.3) | 0.9 (0.8; 1.0) |
|  | Women | 367 | 1,786 | 0.3 (0.3; 0.3) | 1.1 (0.9; 1.2) |
| Bipolar disorder | Men | 146 | 544 | 0.2 (0.1; 0.2) | 1.7 (1.4; 2.0) |
|  | Women | 166 | 670 | 0.1 (0.1; 0.2) | 1.2 (1.1; 1.5) |
| Unipolar depression | Men | 2,889 | 10,021 | 3.1 (3.0; 3.2) | 1.8 (1.8; 1.9) |
|  | Women | 3,628 | 12,119 | 3.1 (3.0; 3.2) | 1.6 (1.5; 1.6) |
| Anxiety disorders | Men | 2,438 | 1,768 | 2.6 (2.5; 2.7) | 8.3 (7.8; 8.9) |
|  | Women | 2,713 | 3,592 | 2.3 (2.2; 2.4) | 3.5 (3.3; 3.7) |
| Posttraumatic stress disorder | Men | 221 | 1,148 | 0.2 (0.2; 0.3) | 1.0 (0.9; 1.2) |
|  | Women | 303 | 1,584 | 0.3 (0.2; 0.3) | 0.6 (0.6; 0.7) |
| Adjustment disorder | Men | 991 | 3,562 | 1.1 (1.0; 1.1) | 1.6 (1.5; 1.8) |
|  | Women | 1,786 | 5,233 | 1.5 (1.4; 1.6) | 1.6 (1.5; 1.7) |
| Neurotic and somatoform disorders | Men | 140 | 531 | 0.2 (0.1; 0.2) | 1.5 (1.2; 1.8) |
|  | Women | 270 | 1,075 | 0.2 (0.2; 0.3) | 1.2 (1.0; 1.4) |
| Behavioral syndromes | Men | 325 | 910 | 0.3 (0.3; 0.4) | 2.1 (1.8; 2.4) |
|  | Women | 375 | 801 | 0.3 (0.3; 0.3) | 2.2 (1.9; 2.5) |
| Personality disorders | Men | 130 | 957 | 0.1 (0.1; 0.2) | 0.7 (0.6; 0.9) |
|  | Women | 189 | 1,085 | 0.2 (0.1; 0.2) | 0.8 (0.7; 0.9) |
| Other | Men | 209 | 1,289 | 0.2 (0.2; 0.3) | 0.9 (0.8; 1.0) |
|  | Women | 291 | 1,213 | 0.2 (0.2; 0.3) | 1.1 (1.0; 1.3) |
| **Psychotropic Medication** | | | | | |
| Any psychotropic medication | Men | 43,261 | 107,472 | 54.2 (53.7; 54.7) | 2.6 (2.6; 2.7) |
|  | Women | 46,904 | 121,432 | 50.4 (49.9; 50.9) | 2.1 (2.0; 2.1) |
| Antipsychotics | Men | 14,313 | 31,317 | 15.4 (15.2; 15.7) | 3.0 (3.0; 3.0) |
|  | Women | 13,170 | 28,472 | 11.2 (11.0; 11.4) | 2.4 (2.3; 2.4) |
| Antidepressants | Men | 27,574 | 76,474 | 32.6 (32.3; 33.0) | 2.3 (2.3; 2.4) |
|  | Women | 30,380 | 89,939 | 29.7 (29.3; 30.0) | 1.7 (1.7; 1.8) |
| Lithium | Men | 64 | 396 | 0.1 (0.1; 0.1) | 1.0 (0.8; 1.3) |
|  | Women | 110 | 520 | 0.1 (0.1; 0.1) | 1.1 (0.9; 1.3) |
| Anxiolytics | Men | 18,130 | 38,281 | 20.5 (20.2; 20.8) | 3.1 (3.0; 3.1) |
|  | Women | 23,353 | 51,446 | 22.0 (21.7; 22.3) | 2.4 (2.3; 2.4) |
| Alcohol and opioid dependence medication | Men | 871 | 4,759 | 0.9 (0.9; 1.0) | 1.1 (1.0; 1.1) |
|  | Women | 613 | 2,871 | 0.5 (0.5; 0.6) | 1.1 (1.0; 1.1) |
| Sensitivity analysis for patients without pre-existing physical comorbidities (Charlson-Comorbidity-Index = 0) to control for the cumulative burden of physical conditions. Incidence rates (IR) and hazard ratios (HR) for mental disorders and psychotropic medication.  ^1^ Any mental disorder requiring a hospital admission and/or psychotropic medication  ^2^ Incidence rate (IR) per 1,000 person years in the cancer cohort  ^3^ Hazard ratio (HR) in the cancer cohort versus comparison cohort | | | | | |

| **Table S7.** Sensitivity analysis including patients with cancer and comparisons with preexisting mental disorders (N = 2,609,014) | | | | | |
| --- | --- | --- | --- | --- | --- |
|  | **Sex** | **n _Cancer_** | **n _Comparison_** | **IR^2^ (95% CI)** | **HR^3^ (95% CI)** |
| **Any mental disorder**^1^ | Total | 229,551 | 885,657 | 97.9 (97.5; 98.3) | 2.1 (2.1; 2.1) |
|  | Men | 103,894 | 378,317 | 94.8 (94.2; 95.4) | 2.3 (2.3; 2.3) |
|  | Women | 125,657 | 507,340 | 100.6 (100.1; 101.2) | 1.9 (1.9; 1.9) |
| **Hospital Admission** | | | | | |
| Any hospital admission for a mental disorder | Men | 25,254 | 104,421 | 17.9 (17.7; 18.2) | 1.9 (1.8; 1.9) |
|  | Women | 30,602 | 124,855 | 16.4 (16.2; 16.6) | 1.7 (1.7; 1.7) |
| Substance use disorder | Men | 10,131 | 51,083 | 7.0 (6.8; 7.1) | 1.5 (1.5; 1.5) |
|  | Women | 7,746 | 34,980 | 3.9 (3.8; 4.0) | 1.4 (1.4; 1.5) |
| Schizophrenia and related disorders | Men | 1,275 | 9557 | 0.9 (0.8; 0.9) | 1.0 (0.9; 1.0) |
|  | Women | 2,040 | 12,197 | 1.0 (1.0; 1.1) | 1.1 (1.1; 1.2) |
| Bipolar disorder | Men | 742 | 4,353 | 0.5 (0.4; 0.5) | 1.3 (1.2; 1.4) |
|  | Women | 1,343 | 7,164 | 0.7 (0.6; 0.7) | 1.3 (1.2; 1.4) |
| Unipolar depression | Men | 8,343 | 36,790 | 5.7 (5.5; 5.8) | 1.8 (1.7; 1.8) |
|  | Women | 13,153 | 60,615 | 6.7 (6.6; 6.8) | 1.6 (1.5; 1.6) |
| Anxiety disorders | Men | 6,110 | 7,676 | 4.1 (4.0; 4.2) | 6.1 (5.9; 6.3) |
|  | Women | 8,117 | 18,514 | 4.1 (4.0; 4.2) | 2.8 (2.7; 2.9) |
| Posttraumatic stress disorder | Men | 584 | 3,706 | 0.4 (0.4; 0.4) | 1.1 (1; 1.2) |
|  | Women | 905 | 5,907 | 0.5 (0.4; 0.5) | 0.7 (0.6; 0.7) |
| Adjustment disorder | Men | 2,524 | 11,785 | 1.7 (1.6; 1.8) | 1.6 (1.5; 1.7) |
|  | Women | 4,809 | 20,231 | 2.4 (2.4; 2.5) | 1.5 (1.5; 1.6) |
| Neurotic and somatoform disorders | Men | 359 | 2,040 | 0.2 (0.2; 0.3) | 1.3 (1.2; 1.5) |
|  | Women | 845 | 4,987 | 0.4 (0.4; 0.5) | 1.1 (1.0; 1.2) |
| Behavioral syndromes | Men | 640 | 2,462 | 0.4 (0.4; 0.5) | 1.9 (1.8; 2.1) |
|  | Women | 900 | 3,023 | 0.5 (0.4; 0.5) | 1.9 (1.8; 2.0) |
| Personality disorders | Men | 478 | 3,741 | 0.3 (0.3; 0.4) | 0.8 (0.8; 0.9) |
|  | Women | 1,028 | 6,671 | 0.5 (0.4; 0.5) | 1.0 (0.9; 1.0) |
| Other | Men | 718 | 5,799 | 0.5 (0.4; 0.5) | 0.9 (0.8; 1.0) |
|  | Women | 1,184 | 7,144 | 0.6 (0.6; 0.6) | 1.1 (1.0; 1.2) |
| **Psychotropic Medication** | | | | | |
| Any psychotropic medication | Men | 97,940 | 354,730 | 87.1 (86.6; 87.7) | 2.3 (2.3; 2.3) |
|  | Women | 121,087 | 491,810­ | 94.7 (94.2; 95.3) | 1.9 (1.9; 1.9) |
| Antipsychotics | Men | 32,692 | 110,314 | 22.7 (22.5; 22.9) | 2.3 (2.3; 2.4) |
|  | Women | 36,998 | 136,490 | 19.5 (19.3; 19.7) | 1.9 (1.9; 1.9) |
| Antidepressants | Men | 66,233 | 261,431 | 53.6 (53.2; 54.1) | 2.1 (2.0; 2.1) |
|  | Women | 85,868 | 380,272 | 57.4 (57.1; 57.8) | 1.6 (1.6; 1.7) |
| Lithium | Men | 597 | 3,991 | 0.4 (0.4; 0.4) | 1.1 (1.0; 1.2) |
|  | Women | 1,177 | 6,821 | 0.6 (0.6; 0.6) | 1.2 (1.1; 1.2) |
| Anxiolytics | Men | 43,640 | 136,666 | 32.6 (32.3; 32.9) | 2.6 (2.5; 2.6) |
|  | Women | 64,744 | 226,941 | 39.9 (39.6; 40.3) | 2.1 (2.1; 2.1) |
| Alcohol and opioid dependence medication | Men | 3,780 | 23,156 | 2.6 (2.5; 2.6) | 1.2 (1.2; 1.2) |
|  | Women | 2,960 | 17,152 | 1.5 (1.4; 1.5) | 1.1 (1.1; 1.2) |
| Sensitivity analysis including patients with pre-existing mental disorders, reflecting a well-known risk factor for subsequent mental disorders. Incidence rates (IR) and hazard ratios (HR) for mental disorders and psychotropic medication.  ^1^ Any mental disorder requiring a hospital admission and/or psychotropic medication  ^2^ Incidence rate (IR) per 1,000 person years in the cancer cohort  ^3^ Hazard ratio (HR) in the cancer cohort versus comparison cohort | | | | | |


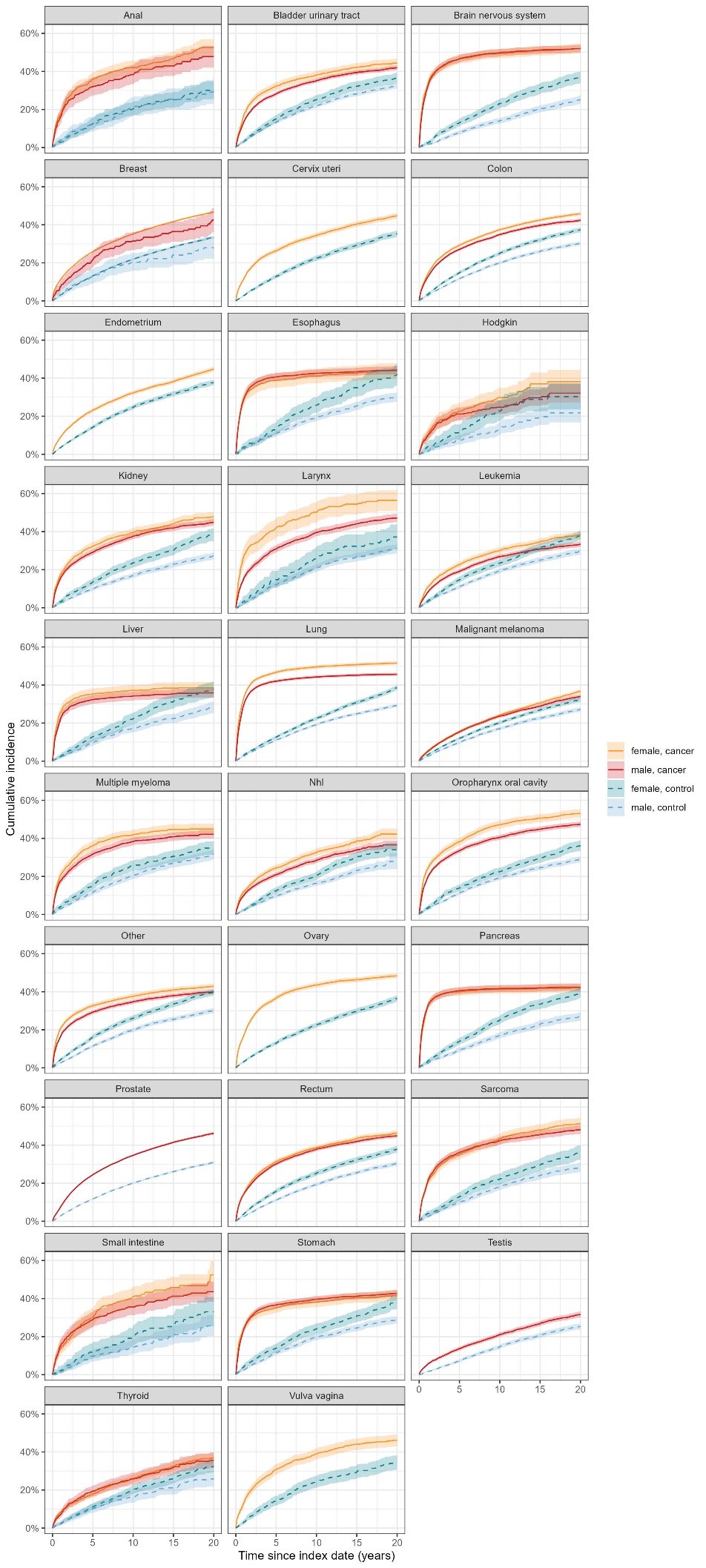

**Figure S1**. Cumulative incidence for any mental disorder, stratified by cancer type
